# Metabolic Dysfunctions of Intestinal Fatty Acids and Tryptophan Reveal Immuno-Inflammatory Response Activation in IgA Nephropathy

**DOI:** 10.3389/fmed.2022.811526

**Published:** 2022-02-03

**Authors:** Hongwei Wu, Donge Tang, Manhua Yun, Haiping Liu, Shaoxing Huang, Chen Yun, Berthold Hocher, Xinzhou Zhang, Fanna Liu, Lianghong Yin, Yong Dai

**Affiliations:** ^1^Clinical Medical Research Center, Guangdong Provincial Engineering Research Center of Autoimmune Disease Precision Medicine, Shenzhen Engineering Research Center of Autoimmune Disease, The Second Clinical Medical College of Jinan University, Shenzhen People's Hospital, Shenzhen, China; ^2^Department of Nephrology and Blood Purification, The First Affiliated Hospital of Jinan University, Jinan University, Guangzhou, China; ^3^Department of Medicine Nephrology, University Medical Centre Mannheim, Heidelberg, Germany; ^4^The Second People's Hospital of Lianping County, Guangdong, China; ^5^Charité -Universitätsmedizin Berlin, Berlin, Germany; ^6^Key Renal Laboratory of Shenzhen, Department of Nephrology, Shenzhen People's Hospital, The Second Clinical Medical College of Jinan University, Shenzhen, China

**Keywords:** IgA nephropathy, fatty acid metabolism, tryptophan metabolism, immune, 3-indolepropionic acid

## Abstract

**Background:**

Immunoglobulin A nephropathy (IgAN) is the most common form of primary glomerulonephritis. Although an important link between intestinal metabolites and immune activity is widely established, the metabolic profile of IgAN is still poorly understood, which severely limits the mechanistic studies and therapy of IgAN.

**Methods:**

The diversity of intestinal flora and relative abundance of metabolites in IgAN patients and healthy subjects were measured by 16s ribosomal RNA gene sequencing combined with liquid chromatography tandem-mass spectrometry. The levels of serum Gd-IgA1, IL-6, IL-10, IL-22, and TNF-a were tested by ELISA. We employed the tryptophan-targeted UHPLC-MRM-MS approach to assess the content of tryptophan metabolites quantitatively.

**Results:**

Intestinal fatty acid levels, mainly unsaturated fatty acids, were observed to be dramatically decreased in IgAN patients. Disorders in linoleic acid and arachidonic acid metabolism, metabolic imbalances of anti-/pro- inflammatory fatty acid metabolites, and intestinal AhR signaling deficiency might reflect the damage of the intestinal mucosal barrier in IgAN patients. In addition, we found that high levels of Gd-IgA1, IL-22, and TNF-α were associated with the activity of the tryptophan-kynurenine metabolic pathway, as well as lower levels of 3-indolepropionic acid. 3-indolepropionic acid, kynurenine, and indoleacrylic acid had synergistic effects on regulating immuno-inflammatory responses in IgAN patients.

**Conclusions:**

The metabolic characteristic of fatty acids and tryptophan in the intestinal system is disturbed in IgAN patients, leading to active immune-inflammatory reactions.

## Introduction

Immunoglobulin A nephropathy (IgAN) is an autoimmune disease with unclear etiology. The aberrant glycosylation of IgA1 is commonly acknowledged to be the initial link in the genesis of IgAN (1). A prominent role of gut microbiota and intestinal metabolites has been established in maintaining the intestinal mucosal barrier function. Also, the gut system is an essential location of immune regulation, maintaining the host immune homeostasis through bacterial surface antigen recognition and secondary metabolite synthesis (2).

Fatty acid and amino acid metabolites produced by gut bacteria have been found to have a substantial impact on the intestinal epithelial barrier and immunity. The metabolites of linoleic acid, such as 10-hydroxy-cis-12-octadecenoic acid and 10-oxo-trans-11-octadecenoic, could ameliorate intestinal epithelial impairment (3, 4). Dietary bioactive fatty acids had suppressive effects on lymphopoiesis and antibody production, lowering IL-1, IL-6, and TNF levels (5). Besides, gut microbiome-derived amino acid derivates were involved in the biological processes of the host immune responses (6). Tryptophan metabolites such as indole, 3-indolepropionic acid, and indole-3-acetic acid are essential aryl hydrocarbon receptors (AhR) that keep the intestinal immune balance and mucosal integrity (7, 8). Therefore, a systematic understanding of the biological role of fatty and amino acid metabolism will provide in-depth insights into the causes of immune dysfunction in IgAN.

The side effects of immunosuppressive drugs and the high relapse rate after therapy are the current challenges in the therapy of IgA nephropathy. The complex pathophysiological process determines the need for comprehensive treatment and management of IgA nephropathy patients. Maintaining metabolic balance through dietary management and nutritional supplementation has been recognized in the treatment of other diseases such as diabetes and cardiovascular disease. Effective nutritional and dietary strategies are still lacking for IgAN patients. Therefore, a comprehensive picture of metabolic profiles in the gut and blood systems will provide better adjuvant treatments for IgAN patients.

## Methods

### Study Design

Stool samples from 15 IgAN patients and 30 healthy controls were examined by 16s ribosomal RNA gene sequencing and liquid chromatography-tandem mass spectrometry (LC-MS/MS) to establish the gut microbes and intestinal metabolic profiles of IgAN patients. Serum samples were collected from the above-mentioned study subjects (15 IgAN patients and 15 healthy controls) and metabolite analysis was performed by LC-MS/MS approach to reveal the metabolic profile of the circulation system in IgAN patients. One patient's blood sample was excluded due to hemolysis, leaving 14 IgAN patients for further analysis. To verify the diagnostic value of 3-indolepropionic acid for IgA nephropathy, we also collected serum samples from patients with diabetic nephropathy (*n* = 26) and membranous nephropathy (*n* = 20) and used LC-MS/MS method to test serum 3-indolepropionic acid levels. In addition, we performed tryptophan-targeted UHPLC-MRM-MS analysis on an additional 15 IgAN patients and 10 healthy subjects to verify the disorder of the tryptophan metabolic pathway in IgAN patients. The clinical parameters of the included subjects are presented in Supplementary Table 1. IgAN patients were diagnosed by histopathology with median estimated glomerular filtration rate (eGFR) > 60 ml/min/1.73 m^2^ (abbreviated MDRD formula). Participants had not been treated with any drugs (e.g., antibiotics or aspirin) or nutritional supplements for at least 2 months. All subjects were Han Chinese from southern China with comparable eating habits and lifestyles. The present study was conducted following the principles of the Declaration of Helsinki and was authorized by the Ethics Board of Shenzhen People's Hospital (LL-KY-2019514).

### LC-MS/MS Analysis

Sample collection: feces samples were put into two frozen tubes and immediately placed on dry ice after collection. Four to six milliliter of 8-h fasting venous blood samples were collected and then centrifuged at 3,000 rpm for 10 min at 25°C. All the supernatants were stored at −80°C freezer.

Metabolites extraction: 400 μL of extraction solution (acetonitrile: methanol = 1: 1, containing 1 μg/mL 2-chloro-L-phenylalanine) was added to 100 μL of each serum sample. The mixed solution was centrifuged at 12,000 rpm for 15 min after sonication in a water bath at 4°C. For fecal samples pretreatment, 50 mg of stool were mixed with 1 mL extraction solution (acetonitrile: methanol: water = 2: 2: 1, containing 1 μg/ml 2-chloro-L-phenylalanine). After vortexing for 30 s, all the samples were homogenized at 45 Hz for 5 min and then sonicated at 4°C for 5 min, followed by centrifugation at 12,000 rpm for 15 min at 4°C. The supernatants were collected and subjected to metabolomics profile analysis by UHPLC-MS. Quality control samples were prepared by mixing an equal volume (10 μL) of each serum or fecal supernatant.

Chromatographic separation of the target compounds was performed through a Waters ACQUITY UPLC BEH Amide (2.1 × 100 mm, 1.7 μm) in the UHPLC system (Agilent Technologies, California, USA) equipped with Thermo Q Exactive HFX mass spectrometer (Vanquish, Thermo Fisher Scientific, USA). The liquid chromatography included 2 phases: mobile phase A was 0.1% formic acid in water (positive ion mode) or 5 mmol/L ammonium acetate in water (negative ion mode) and mobile phase B was acetonitrile. The elution gradient was used as follows: 1% B, 1 min; 99% B, 8 min; 99% B, 10 min; 1% B, 1 min; 1% B, 12 min. The injection volume was 3 μL. The flow rate was set to 0.5 mL/min. ESI source conditions were set as follows: spray voltage: 4.0 kV (positive mode) or −3.6 kV (negative mode); Aux gas flow rate: 15 Arb; sheath gas flow rate: 45 Arb; capillary temperature: 400°C.

Data preprocessing and annotation: the MS raw data were converted to mzXML format and then processed for peak identification, extraction, alignment, and integration. After filtering the relative standard deviation of the peaks >30% in the quality control samples, we then compared the remaining peaks to the retention time and mass to charge ratio (*m*/*z*) indexes in the online database of HMDB and KEGG, thus generating an initial data matrix. Peaks with missing values in over 50% of samples in this data matrix were further removed. We used the half of minimum value to fill up the rest of the missing values and normalized the data to the peak intensity of the internal standard.

Principal component analysis was performed to assess the difference in metabolomics profile between groups. To effectively determine the universal variation trends in a class of bioactive metabolites and explain the overall changes in a specific metabolic pathway, we set up the absolute value of fold-change > 1.5, VIP > 1, and *P* < 0.05 as screening thresholds to identify differential metabolites. MetaboAnalyst 5.0 was utilized for the statistical analysis of the secondary metabolites and pathway enrichment.

### Tryptophan-Targeted UHPLC-MRM-MS Analysis

Standard solution preparation: we prepared 1 mmol/L individual stock solutions of each tryptophan metabolite by diluting each standard substance (Supplementary Table 2). An aliquot of each stock solution was combined to form a mixed working standard solution, which was then gradually diluted to prepare a series of standard calibration solutions.

UHPLC-MRM-MS Analysis: we used EXIONLC System equipped with a Waters ACQUITY UPLC HSS PFP column (100 × 2.1 mm, 1.8 μm, Waters) to perform the UHPLC separation. The liquid chromatogram included mobile phase A (0.1% formic acid in water) and mobile phase B (0.1% formic acid in acetonitrile). The column temperature was 40°C, and the auto-sampler temperature was 4°C. The injection volume was 10 μL. A SCIEX 6500 QTRAP+ triple quadrupole mass spectrometer (Sciex) was employed for assay development. An ion source was used with the following parameters: ionSpray voltage = ±4,500 V, curtain gas = 40 psi, temperature = 500°C, ion source gas 1 = 30 psi, ion source gas 2 = 30 psi. The collision energy of each Q1/Q3 pair was optimized by several of the most sensitive transitions used in the MRM scan mode (Supplementary Table 2).

Calibration curves: calibration solutions were subjected to UPLC-MRM-MS/MS analysis. The calibration parameters of tryptophan metabolites and the detailed calibration curves for each of the analyte are listed in Supplementary Table 2. We used the least-squares method to perform regression fitting. 1/ × weighting was applied in the curve fitting. If the calibration accuracy was not within 80–120%, the level was eliminated from the calibration.

Limit of detection (LOD) and limit of quantitation (LOQ): before UHPLC-MRM-MS analysis, the calibration standard solution was diluted stepwise with a dilution factor of 2. The lower limits of detection (LLODs) and lower limits of quantitation (LLOQs) were calculated using the signal-to-noise ratios (S/N). The metabolite concentrations that led to peaks with S/N of 3 and 10 were referred to as LLODs and LLOQs, respectively. The quantitative parameters of the target compound and the analytical recoveries and relative standard deviations of the QC samples are displayed in Supplementary Table 2. The percent recovery was calculated as [(mean observed concentration)/(spiked concentration)] × 100%. The analysis metrics revealed that the methodology used herein allowed for accurate quantitation of the targeted metabolites in biological samples.

### 16s rRNA Sequencing

Genomic DNA from feces samples was extracted using PowerSoil DNA Isolation Kit (Qiagen, Hilden, Germany). We used universal primers (forward: 5'-ACTCCTACGGGAGGCAGCA-3'; reverse: 5'-GGACTACHVGGGTWTCTAAT-3') to amplify the V3 and V4 regions of the 16s rRNA gene, after finishing DNA quality and quantity by NanoDrop spectrophotometry (NanoDropo, Germany). PCR fragments were purified using MPure XP beads (Beckman Coulter, Indiana, USA) and then sequenced using the Illumina HiSeq platform (Illumina, California, USA). Paired-end reads obtained by Illumina sequencing were merged into specific tags based on their overlapping information by FLASH software (v1.2.7). After removing the low-quality tags by Trimmomatic software (v0.33), we used UCHIME v4.2 to filter the chimeric sequences of the clean tags (9).

High-quality tags with a similarity > 97% were allocated to an operational taxonomic unit (OTU) using USERACH software (v10.0). OTU was filtered with a threshold of 0.005% of all sequence numbers and taxonomically annotated based on the Silva database (www.Arb-silva.de). We used the DRP classifier (v2.2) to classify the OTU into different phylogenetic levels (Phylum, class, order, family, genus, species). Furthermore, Linear discriminant analysis coupled with effect size (LEfSe value > 2) and Wilcoxon ran-sum test (*P* < 0.05) were used to identify significantly changed microorganisms between groups.

### Enzyme-Linked Immunosorbent Assay Testing

Serum levels of Gd-IgA1, IL-22, IL-10, IL-6, and TNF-a were tested by individual commercial ELISA kits (Fine Biotech Co., Ltd., Wuhan, China). All the steps were conducted according to the operations manual (available online: https://www.fn-test.com/). Briefly, six standard holes, two blank control holes, and the remaining sample wells were set on the enzyme-labeled plate. The experiment was repeated twice for each sample. After incubation, washing, coloration, incubation, and termination, we measured the absorbance of each well at a wavelength of 450 nm and plotted standard curves.

### Statistical Analysis

The student's *t*-test was applied to identify changed metabolites between the IgAN and HC groups. ANOVA analysis was used to compare metabolite levels across multiple experimental groups. Laboratory indexes were compared using Wilcoxon rank-sum test. Relationships between the intestinal flora, serum and fecal metabolites, and the clinical indexes were evaluated by Spearman's correlation analysis. Fisher's exact test was adopted to test the significantly altered metabolites class in the serum and fecal samples. Plots were done using R programming, GraphPad Prism 7.0, Adobe Illustrator CS4, Cytoscape, and online website: https://www.omicstudio.cn/index.

## Results

### The Characteristics of the Intestinal Metabolic Landscapes in IgAN Patients

**Figure 1:**
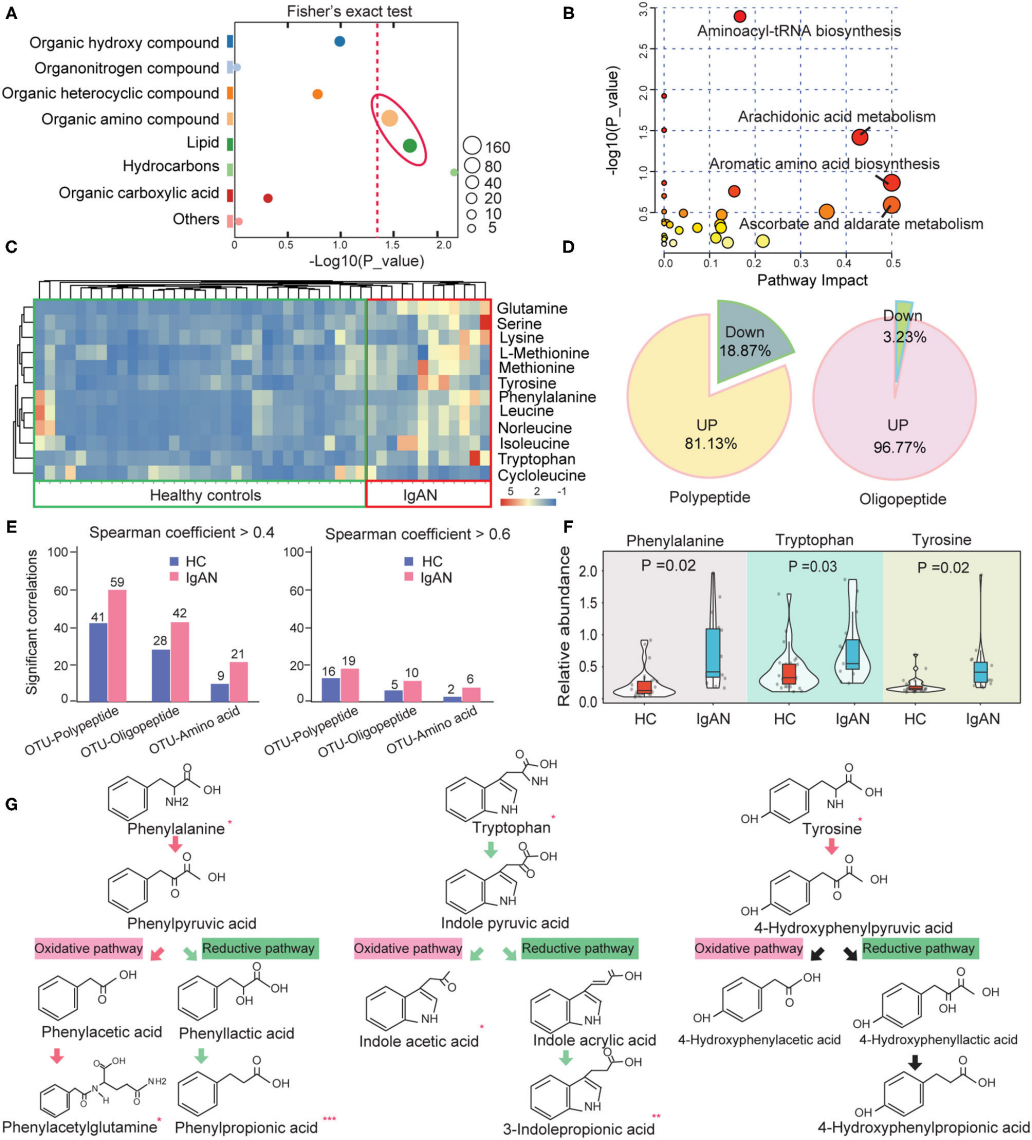
Changes in the abundance of intestinal amino acids and their derivatives in IgAN. **(A)** Fisher's exact test analysis of significantly altered metabolites class in fecal samples. The size of each spot indicates the number of metabolites that underwent significant changes in the corresponding metabolites class. The dashed red line indicates the significance level (*P* < 0.05). **(B)** Enrichment diagram of the intestinal metabolic pathways. The horizontal axis and the bubble size indicate the impact of metabolic pathways, whereas the vertical axis and the bubble color represent the *P*-value effect size. **(C)** Heatmap of the significantly changed amino acids in the intestinal metabolic system. **(D)** Pie chart showing the proportion of up- and downregulated peptides and oligopeptides in the gut of IgAN patients. **(E)** Statistic analysis results of the number of pairs with significant correlations between OTUs and peptides, oligopeptides, and amino acids in the feces of the healthy controls and IgAN patients. Left: Spearman rank coefficient > 0.4 and *P* < 0.05; Right: Spearman coefficient > 0.6 and *P* < 0.05. **(F)** Comparison of the relative abundance of fecal aromatic amino acids between the IgAN and HC groups. **(G)** Comparison of the abundance of the metabolites in aromatic amino acid metabolism between the two groups. The arrows indicate the direction of oxidation or the reduction pathway of amino acids, with red arrows denoting upregulated metabolic pathways and green arrows representing the downregulated metabolic pathways. ^*^Represents a significant variation in the metabolite abundance among the groups. ^*^P < 0.05; ^**^P < 0.01; ^***^P < 0.001.

Based on the LC-MS/MS database, we discovered 843 secondary metabolites in the ESI+/ESI– model. These metabolites were then classified into eight main chemical categories. Organic ammoniates (40%) dominated the intestinal metabolites, followed by lipids (16.1%) and organic hydroxyl compounds (12.3%) (Supplementary Table 3). In IgAN patients, 101 metabolites were significantly elevated and 111 metabolites were significantly downregulated (Supplementary Figures 1A,B; Supplementary Table 4). Organic ammoniates (48.1%), including amino acids and derivatives, and lipids (23.1%), accounted for the majority of the changed metabolites (Supplementary Figure 1C). Fisher's exact test showed that changes in organic ammoniates and lipids were remarkable (Figure 1A; Supplementary Table 5). The pathway enrichment analysis showed that aminoacyl-tRNA biosynthesis, arachidonic acid metabolism, and aromatic amino acid biosynthesis were the most significantly changed pathways in IgAN patients. These findings suggested that the intestinal metabolic landscapes of IgAN patients were characterized by the disorder of amino acid and lipid metabolism (Figure 1B).

### Features of the Intestinal Amino Acid Metabolism in IgAN Patients

As previously stated, the metabolic dysfunction of intestinal amino acids was conspicuous in IgAN patients. The relative abundance of these significantly changed amino acids is shown in Figure 1C. Surprisingly, except for cycloleucine, the remaining amino acids showed an upward trend in the IgAN group. A rising tendency in the levels of oligopeptides and polypeptides was also found in the IgAN group (Figure 1D), suggesting that gut bacteria performed extensive proteolysis in IgAN patients. To validate these findings, we analyzed correlations between the optical transform unit (OTU) of gut bacteria and that of the products of protein hydrolysis using Spearman correlation. The resulted showed that IgAN patients had more correlated pairings between OUT and proteolytic products based on the screening criteria as a *P*-value < 0.05 and *r* > 0.4 (Figure 1E), indicating that changes in protein metabolism in IgAN patients were linked to gut microbiota activity. Among these proteolytic products, IgAN patients have significantly increased levels of aromatic amino acids, such as phenylalanine, tryptophan, and tyrosine (Figure 1F). Interestingly, the concentrations of oxidation products of phenylalanine (phenylpyruvic acid and phenylacetylglutamine) increased significantly in IgAN patients, whereas the reduced product (phenylpropionic acid) showed the reverse tendency. In addition, we observed decreased levels of the oxidation and reduction products of tryptophan (Figure 1G). The reduced levels of these AhR ligands in the intestinal system, including indoleacetic acid and 3-indolepropionic acid, may be suggestive of damage to the intestinal barrier in IgAN patients (10), which will be further analyzed below.

### Features of the Intestinal Lipid Metabolism in IgAN Patients

**Figure 2:**
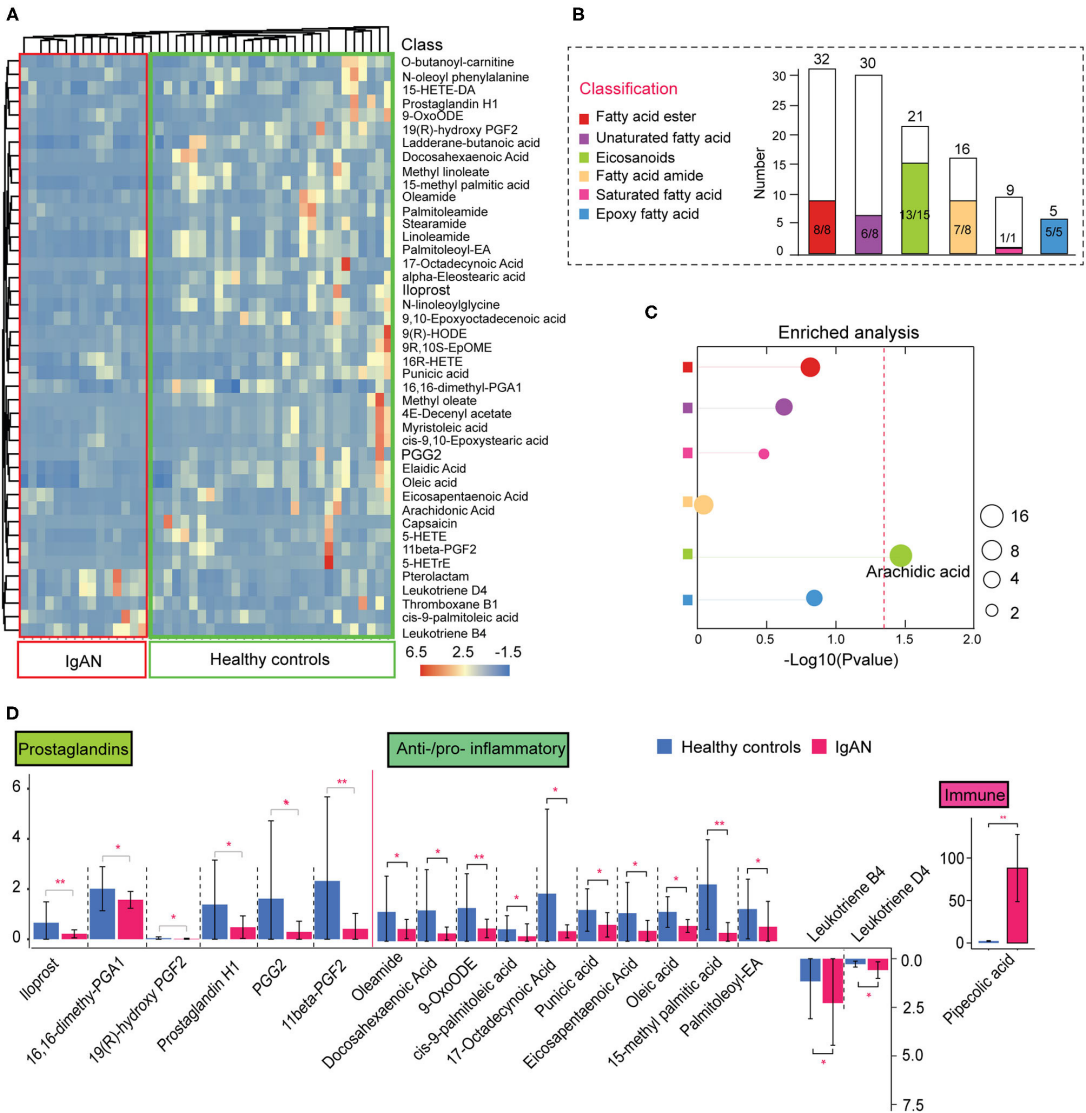
Alterations in intestinal lipid metabolism in IgAN. **(A)** Heatmap of the significantly changed lipid metabolites in the feces samples. **(B)** Histogram of the overall variation trends in different lipid classifications. The height of the bar chart (white) indicates the number of all metabolites identified by LC-MS/MS in that classification. The colored bar part of the graph represents the number of the significantly altered metabolites under this classification. The denominator indicates the total number of the changed metabolites under this classification and the numerator indicates the number of significantly downregulated metabolites. **(C)** Enrichment analysis of significantly altered metabolites classes in fecal samples. The size of each spot indicates the number of metabolites that underwent significant changes in the corresponding metabolites class. Differently colored circles represent different lipid classifications. The dashed red line indicates the significant level (*P* < 0.05). **(D)** Functional classification of lipid metabolites and comparative analysis of the differences among the groups. ^*^P < 0.05. ^**^P < 0.01.

In addition to the abnormal amino acid metabolism, disruption of intestinal lipid homeostasis was a striking feature of IgAN patients (Figure 1A). Figure 2A depicts the relative abundance of the significantly changed lipid metabolites. Fatty esters accounted for ~16.3% of the total lipid metabolites detected, followed by fatty acid amides (16.3%), eicosanoids (30.6%), epoxy fatty acids (10.2%), unsaturated fatty acids (16.3%), and saturated fatty acids (2%) (Figure 2B). Surprisingly, lipid metabolites showed a general downward trend in IgAN patients, markedly different from the trend in amino acid metabolism. Down-regulated lipid metabolites accounted for ~90.7% of the total differential lipid metabolites. Fisher's exact test showed that changes in arachidonic acid metabolites were remarkable (Figure 2C; Supplementary Table 5). These lipid metabolites were further classified based on their characteristics and relative functions. We found that intestinal barrier protective factors (Prostaglandin metabolites) were sharply reduced in IgAN patients (11). Moreover, anti-inflammatory unsaturated fatty acids, such as palmitoleic acid (12), oleic acid (13), and 9-OxoODE (14), were remarkably decreased, whereas pro-inflammatory eicosanoids such as leukotriene B4 and leukotriene D4 (15) were significantly increased (Figure 2D). These findings suggested an imbalance between anti- and pro-inflammatory metabolites in the gut system of IgAN patients. Notably, increased levels of pipecolic acid, a product of lysine metabolism in activating and promoting immune responses through free radical production (16, 17), were observed in IgAN patients (Figure 2D).

**Figure 3:**
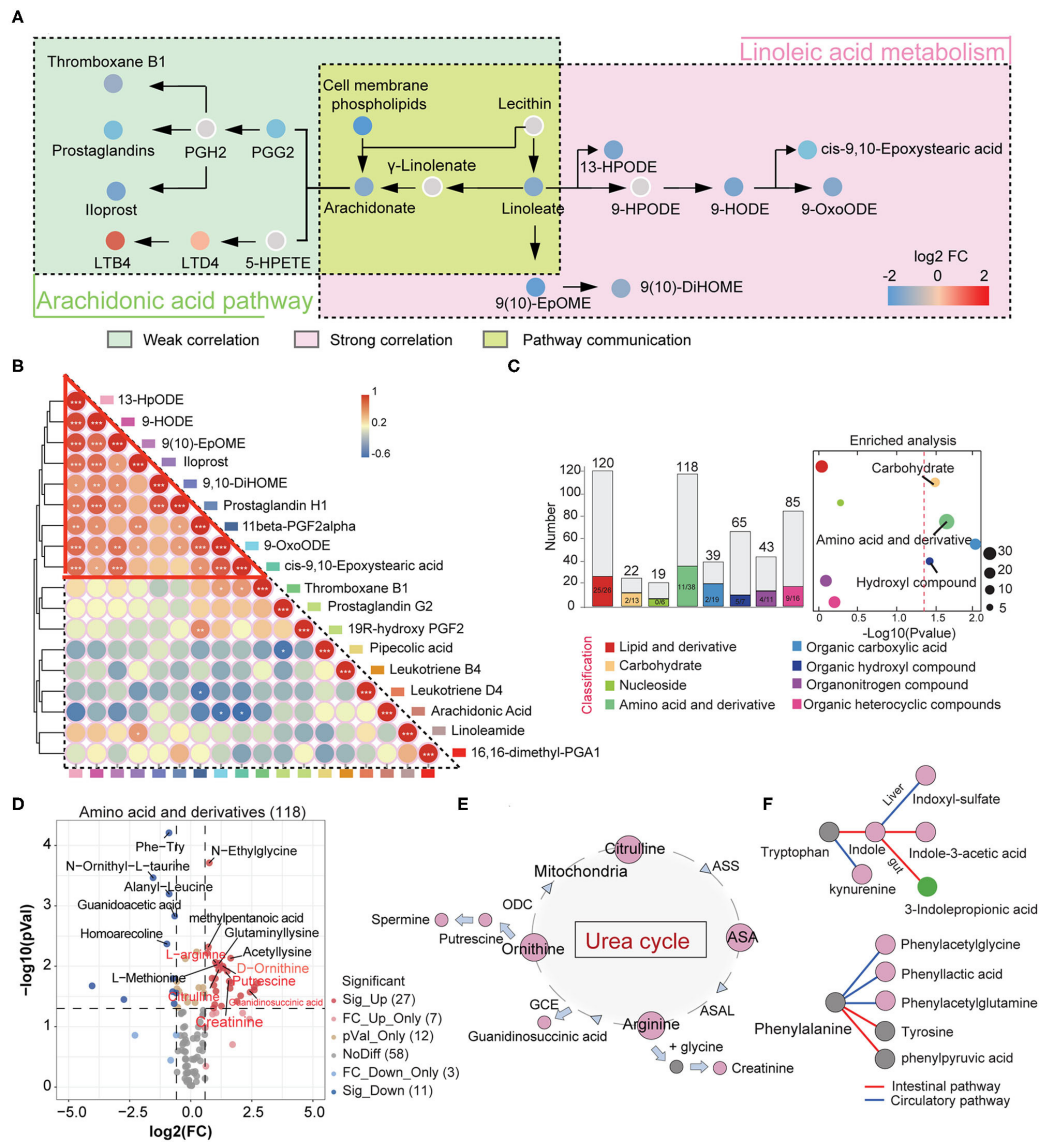
Changes in intestinal linoleic acid metabolic pathways and blood circulating amino acid metabolic pathways in IgAN. **(A)** The left part shows the arachidonic acid metabolic pathway, with the green box indicating weak Spearman's correlation between metabolites on this pathway. The right part represents the linoleic acid oxidation metabolic pathway, with the red box indicating strong correlations between metabolites on this pathway. The yellow area indicates the conversion processes of linoleic acid to arachidonic acid. Each dot represents a metabolite, and the color indicates the degree of alteration in that metabolite. The red color indicates that this metabolite is upregulated in the IgAN group and the blue indicates downregulation. **(B)** Correlation analysis between metabolites identified in the linoleic and arachidonic acids metabolic pathways. Differently colored boxes on the border represent different metabolites. The color of the circles indicates the magnitude of Spearman's correlation coefficient. Changes in color from blue to red indicate a gradual increase in Spearman's correlation. **(C)** Left: Histogram of overall variation trends in different serum metabolite classifications. Right: Enrichment analysis of significantly altered metabolites classes in fecal samples. A detailed description can be found in Figures 2B,C. **(D)** Volcano diagram depicting the intergroup change in the serum amino acids and their derivatives in IgAN. **(E)** Changes in the metabolic processes of the urea cycle in IgAN. Red circles indicate significantly increased expression of this metabolite in the IgAN group compared with the HC group (FC > 2 and *P* < 0.05). The arrows represent the direction of the metabolic process. **(F)** Changes in the metabolic processes of the serum tryptophan and phenylalanine in IgAN. The red circles indicate significantly increased expression of this metabolite and the green circle denotes a significant decrease in the IgAN group. The gray circles indicate statistically insignificant changes.

We speculate that the imbalance in the production of anti- and pro-inflammatory metabolites may result from the disorder of the arachidonic acid metabolic pathway. Therefore, we focused on characterizing changes in arachidonic acid and linoleic acid pathways in IgAN patients. We found that epoxy metabolites in the linoleic acid pathway, such as 9-HPODE, 9-HODE, 9-OxoODE, and 9(10)-EpOME, were significantly reduced in IgAN patients (Supplementary Figure 2A), suggesting that linoleic acid metabolism in the intestinal environment was inhibited. Linoleic and conjugated linoleic acid are essential protective metabolites that regulate host immune and inflammation (18, 19). We also discovered decrease levels of arachidonic acid in the intestinal environment, which might be attributed to a weakened conversion of linoleic acid and phospholipids to arachidonic acid (20) (Supplementary Figure 2B). Importantly, arachidonic acid metabolism increased in the direction of leukotriene metabolism (pro-inflammatory direction), whereas it decreased in prostaglandin metabolism (anti-inflammatory direction) (Figure 3A). Spearman's correlation analysis showed a strong correlation between the metabolites of the linoleic acid pathway, which reflected an overall dysregulation of linoleic acid metabolism in IgAN patients (Figure 3B; Supplementary Table 6). Moreover, we found that some intestinal flora, such as the phylum Bacteroidetes and Faecalibacterium, were associated with changes in these metabolites (Supplementary Table 6).

### The Metabolic Characteristics of the Blood Circulatory System in IgAN Patients

Intestinal flora can influence blood circulation metabolism by metabolizing nutrients and regulating intestinal metabolite levels. Therefore, we characterized the metabolic profile of the blood circulation in IgAN patients and established a communication network between the blood and intestinal metabolic systems. OPLS-DA results suggested that IgAN patients have their distinctive blood circulation metabolic profile (Supplementary Figure 3A).

A total of 664 secondary serum metabolites were discovered. The proportions of these classified metabolites are shown in Supplementary Figure 3B. We obtained 88 significantly upregulated and 59 downregulated metabolites in IgAN patients (Supplementary Table 7). Fisher's exact test showed significant changes in serum amino acids and their derivatives, carbohydrates, and carboxylic acids. These metabolites were predominantly upregulated in IgAN patients (Figure 3C). There was a general downward trend in serum anti-inflammatory fatty acids, which was consistent with the trend found in the gut (Supplementary Figure 3C).

### Characteristics of Serum Amino Acid Metabolism in IgAN Patients

A total of 38 significantly altered amino acids and their derivatives were identified in the circulation system. Twenty-seven amino acids metabolites showed an uptrend in IgAN patients, which was consistent with the trend found in the intestinal system (Figure 3D). Among these metabolites, we observed increased levels of citrulline, arginine, ornithine, and their corresponding downstream metabolites such as creatinine, guanidinosuccinic acid, and putrescine in IgAN patients, suggesting that the urea cycle activation was a key characteristic of IgAN (Figure 3E) (21). Spearman correlation analysis also showed close correlations between urea cycle metabolites (*r* > 0.6, *P* < 0.01) (Supplementary Figure 3D). Notably, enteric-derived toxins (e.g., indoxyl-sulfate and phenylacetylglutamine) generated by aromatic tryptophan and phenylalanine metabolism were also dramatically increased in the blood circulation (22, 23) (Figure 3F; Supplementary Figure 3E), implying that the intestinal mucosal barrier was dysregulated in IgAN patients, resulting in the accumulation of enteric-derived toxins in the organism. The conversion of tryptophan to kynurenine was also enhanced in IgAN patients (Figure 3F). The accumulation of kynurenine in the circulation system might be associated with reduced renal function, but it was more likely a sign of abnormal kynurenine pathway activation caused by immune dysregulation and chronic inflammation in the host (24). Besides, 3-indolepropionic acid, a gut-derived metabolite of tryptophan, was significantly downregulated in the gut and blood systems (Figures 3F, 1F), in contrast to the trend for other metabolites in the tryptophan metabolic pathway, which we believe correlates with decreased levels of intestinal Bacteroidetes (Supplementary Figure 3F).

### Relationship Between Amino Acid Metabolism and Immuno-Inflammatory Response

**Figure 4:**
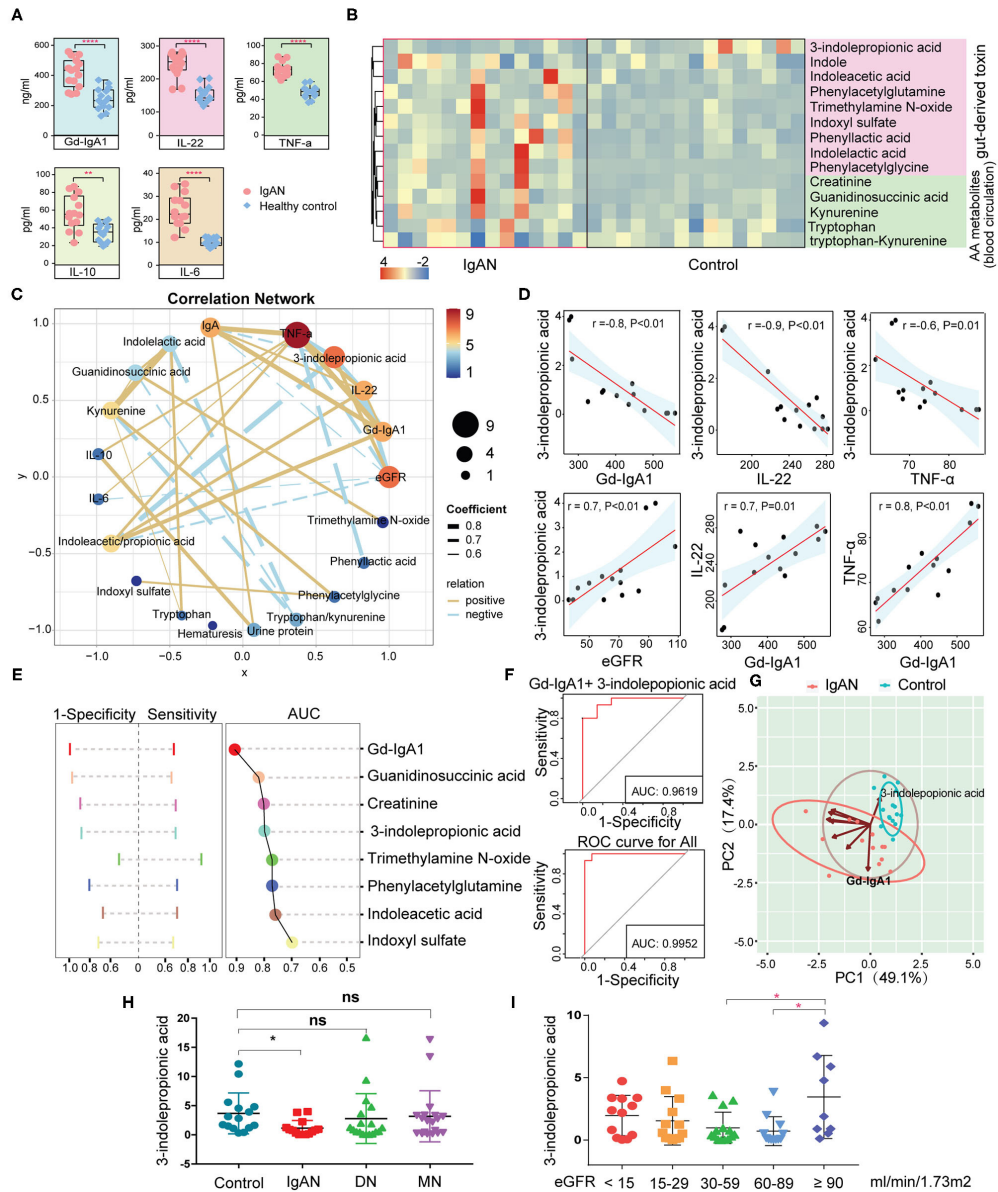
Analysis of the correlation between the circulating amino acid metabolites and the clinical indicators. **(A)** Differences in serum Gd-IgA1, IL-6, IL-10, IL-22, and TNF-a concentrations between the IgAN and the HC groups. **(B)** Heatmap showing the abundance of enteric-derived and circulating amino acid metabolites between the two groups. **(C)** Network diagram depicting Spearman's correlations between amino acid metabolites, clinical indicators (eGFR, 24-h urine protein, and hematuresis), and immune-inflammatory factors (Gd-IgA1, IL-6, IL-10, IL-22, and TNF-a). Pairs with Spearman's coefficient |*r*| > 0.6 and *P* < 0.05 are showed in the network. The solid yellow line indicates positive correlations, whereas the dashed blue line represents negative correlations. Thicker lines refer to stronger relationships. **(D)** Scatter plot reflecting the degree of linear correlation between the two variables. High correlation: 0.7 ≤ |*r*| < 1; Moderate correlation: 0.4 ≤ |*r*| < 0.7. **(E)** ROC graph for the determination of the diagnostic value of amino acid metabolites for IgAN. Left: The range of sensitivity and specificity of each metabolite; Right: The size of the AUC area. **(F)** Logistic regression using the metabolites identified in graph **(E)** to determine the combined diagnostic effect. **(G)** Principal component clustering using the amino acid metabolites in **(E)** as parameters. Coordinate axes PC1 and PC2 are the first and second principal components, i.e., the explanatory rate of the variance by the latent variable. The blue circles indicate healthy control samples, and the red circles refer to IgAN patients. The oval boxed areas indicate grouping by default at a 68% confidence interval. The arrows represent selected amino acid metabolite variables and their directions indicate the correlation between metabolite variables and the principal components. The length of the arrow denotes the magnitude of the contribution. **(H)** Relative abundance of 3-indolepropionic acid in the healthy, IgAN, MN, and DN groups. **(I)** Relative abundance of 3-indolepropionic acid in different eGFR subgroups. ^*^P < 0.05; ^**^P < 0.01; ^****^P < 0.0001; ns, statistically non-significant; ROC, receiver operator characteristic curve; AUC, area under the curve; IgAN, IgA nephropathy; HC, healthy controls; MN, membranous nephropathy; DN, diabetic nephropathy; eGRF, estimated glomerular filtration rate.

The results described above showed aberrant amino acid metabolic pathways in IgAN patients, particularly in the tryptophan metabolism and an accumulation of intestinal-derived toxins created by amino acid metabolism. To analyze the correlation between the amino acid metabolites and the immuno-inflammatory response in IgAN patients, we measured the levels of Gd-IgA1, IL-6, IL-10, IL-22, and TNF-α by ELISA. IgAN patients had significantly higher levels of Gd-IgA1, IL-6, IL-10, IL-22, and TNF-α than healthy subjects (Figure 4A). Based on the different sources of the amino acids, we classified amino acids and their derivatives into two categories: gut-derived amino acid metabolites (e.g., indole, indoxyl-sulfate, and trimethylamine N-oxide) and endogenous amino acid metabolites (e.g., kynurenine and guanidinosuccinic acid) (25). Most amino acids and their derivatives, except for 3-indolepropionic acid, were significantly enhanced in the circulation system of IgAN patients (Figure 4B). Spearman correlation analysis suggested that gut-derived toxins indoxyl-sulfate and trimethylamine N-oxide had no effects on the expression of Gd-IgA1, IL-6, IL-10, IL-22, and TNF-a, whereas 3-indolepropionic acid was inversely related to Gd-IgA1, IL-22, and TNF-a, and positively associated with eGFR (Figures 4C,D). IL-10 showed a positive relationship with 24-h urine protein, while IL-6 was inversely related to eGFR. Besides, positive correlations were found between Gd-IgA1, IL-22, and TNF-a, suggesting a mutually reinforcing connection between immunity and inflammation (Figures 4C,D). Although previous studies have reported that high levels of 3-indolepropionic acid could suppress inflammation and immune response, the role of 3-indolepropionic acid on IgAN remains unknown. We then used AUC analysis to investigate the diagnostic value of these differential amino acid metabolites for IgAN. The results suggested that Gd-IgA1 had the highest diagnostic value for IgAN (AUC = 0.91), followed by guanidinosuccinic acid (AUC = 0.82), creatinine (AUC = 0.81), and 3-indolepropionic acid (AUC = 0.80) (Figure 4E). When several indicators were integrated using logistic regression, the AUC value for the combined diagnosis of Gd-IgA1 and 3-indolepropionic acid was 0.96. The AUC value for all of these differential amino acid metabolites was 0.995 (Figure 4F). PCA clustering with these amino acid metabolites showed a clear separation of samples between the IgAN and HC groups (Figure 4G), suggesting that variation in these amino acid was a prominent feature of IgAN. To test the specificity of 3-indolepropionic acid for the diagnosis of IgAN, we compared 3-indolepropionic acid levels in patients with IgAN, diabetic nephropathy, and membranous nephropathy. We found that serum 3-indolepropionic acid levels were not significantly different in patients with diabetic nephropathy and membranous nephropathy compared to healthy controls, whereas they were considerably lower in IgAN patients (Figure 4H). Considering that 3-indolepropionic acid levels were associated with eGFR in IgAN patients (Figure 4D), we divided these CKD patients into different groups according to their eGFR levels. The results showed that 3-indolepropionic acid decreased significantly in CKD patients with eGFR ranging from 30 to 89 ml/min/1.73 m^2^, while it started to accumulate in the blood circulation system when eGFR was <30 ml/min/1.73 m^2^, although this uptrend did not reach statistical significance (Figure 4I). The relationships between 3-indolepropionic acid and the renal excretory function still need to be further elucidated.

### Alterations in the Circulating Tryptophan Metabolic Pathways in IgAN

**Figure 5:**
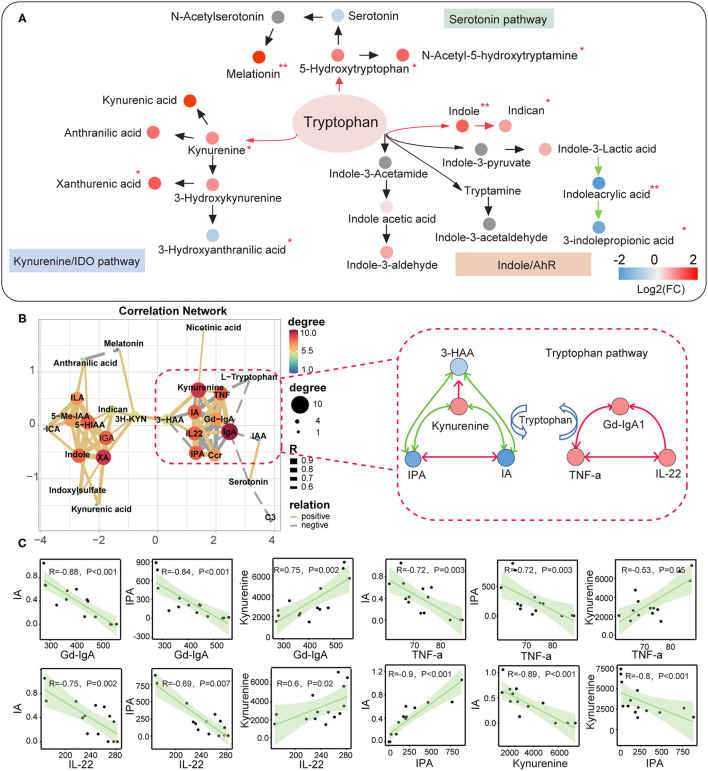
Correlation between the metabolites of the tryptophan metabolic pathway and the clinical indicators of IgA nephropathy based on targeted metabonomics. **(A)** Schematic diagram showing the endogenous tryptophan metabolic pathways and the intestinal tryptophan metabolic pathway. The arrows indicate the metabolic direction. The green arrows indicate the downregulated processes, whereas the red arrows represent the upregulated processes. Each circle represents a metabolite. The circle's color indicates the size of the fold-change value (IgAN vs. HC group) for that metabolite. ^*^Represents a significant variation in the metabolite abundance among the groups. ^*^P < 0.05; ^**^P < 0.01. **(B)** Left: Network diagram showing Spearman's correlations between each tryptophan metabolite, Gd-IgA1, IL-22, and TNF-a (*r* > 0.6 and *P* < 0.05). Right: Schematic diagram showing the interaction of serum kynurenine, IA, and IPA, and their regulatory role in the immune response and inflammation in IgAN. The green lines indicate negative correlations between the two metabolites, and red lines denote positive correlations. **(C)** Scatter plot reflecting the degree of linear correlation between the two variables. IA, indole acrylic acid; IPA, 3-Indolepropionic acid; 3-HAA, 3-Hydroxyanthranilic acid.

We used the targeted metabolomics approach to quantify the circulating metabolites in the tryptophan metabolic pathway since we observed abnormalities in the tryptophan-derived secondary metabolites in IgAN patients. A total number of 24 tryptophan metabolites were identified (Supplementary Figures 4A,B; Supplementary Table 8). Upregulation of indole, 3-hydroxyanthranilic acid, xanthurenic acid, kynurenine levels, and downregulation of 3-indolepropionic acid, indoleacrylic acid, anthranilic acid levels were remarkable features in IgAN patients (Supplementary Figures 4C,D). Metabolites in the endogenous tryptophan metabolic pathway, including the 5-hydroxytryptophan and kynurenine metabolic processes, were generally elevated in IgAN patients, which indicated the 5-hydroxytryptophan and kynurenine metabolic pathways were activated in IgAN patients (Figure 5A). In addition, levels of 3-indolepropionic acid were significantly decreased in IgAN patients. Notably, we also found correlations between gut-derived indole derivatives and metabolites of the endogenous kynurenine pathway, with the most powerful associations among kynurenine, 3-indolepropionic acid, and indoleacrylic acid. Importantly, these three metabolites were significantly associated with the expression of Gd-IgA1, IL-22, and TNF-a (Figures 5B,C; Supplementary Table 9), which suggested that interactions between kynurenine, 3-indolepropionic acid, and indoleacrylic acid might be involved in the regulation of the immuno-inflammatory response in IgAN patients. In addition, although IL-6 showed a significant relationship with indole, no pronounced correlation was found between IL-6 and indole-derived secondary metabolites (Supplementary Figure 5; Supplementary Table 9).

### Gut-Blood Circulation Metabolic Network in IgAN Patients

**Figure 6:**
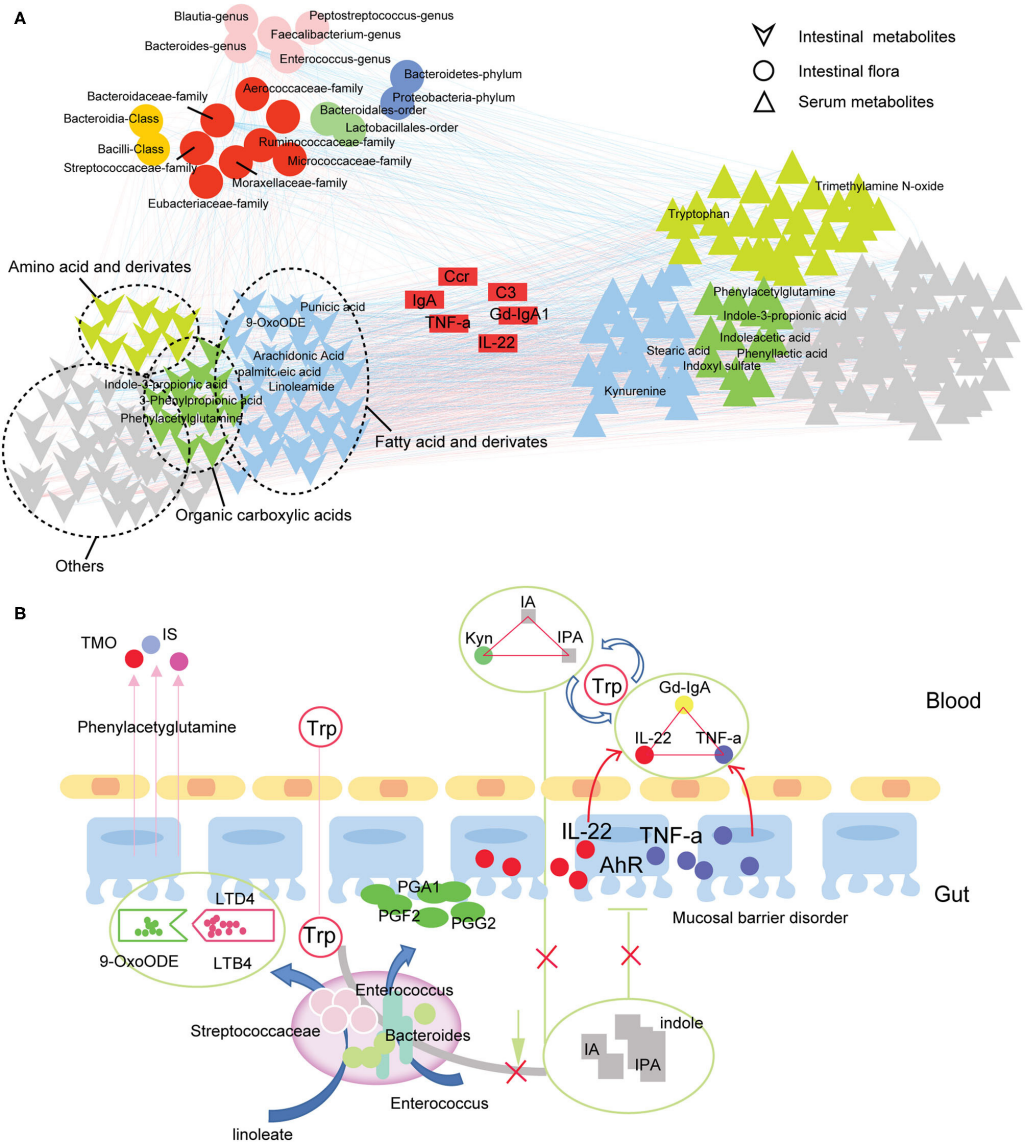
Gut-blood circulation metabolic network in IgAN. **(A)** Correlation network for the intestinal flora, serum metabolites, and fecal metabolites in IgAN patients. The pairs with Spearman's rank correlation |*r*| > 0.6 and *P* < 0.05 are displayed in this network. The circles represent the significantly changed gut flora detected in IgAN patients. Different colors represent different species levels. The inverted triangle (bottom-left) refers to the differential metabolites in the feces samples. The green triangle indicates organic carboxylic acid metabolites, yellow indicates amino acids and their derivatives, blue denotes fatty acids and derivatives, and gray represents other types of metabolites. The positive triangle (bottom-right) represents differential metabolites in serum samples. The blue lines indicate a negative correlation, whereas the red lines denote a positive correlation. **(B)** Communication graph between the intestinal flora, intestinal metabolism, and circulatory metabolism in IgAN patients. The arrows indicate the direction of the metabolism or movement of substances. The solid line indicates a correlation between two substances. IA, indole acrylic acid; IPA, 3-Indolepropionic acid; Kyn, kynurenine; Trp, tryptophan; TMO, trimethylamine N-oxide; IS, indoxyl sulfate; AhR, aryl hydrocarbon receptor.

In this part, we focus on the effect of intestinal flora on metabolites. Spearman correlation analysis revealed strong correlations between gut flora, serum metabolites, and fecal metabolites in IgAN patients (Figure 6A; Supplementary Table 10), showing that the gut and blood circulation systems do not exist as a separate system. Based on these findings, we constructed a communication network between microbiota, fecal metabolites, and serum metabolites in IgAN patients (Figure 6B). As shown in the figure, disturbance in the tryptophan metabolic pathway caused by changes in microbiota levels, particularly reduced quantity of Bacteroidetes, resulted in a decrease in protective AhR ligands (e.g., 3-indolepropionic acid) in the gut system. In addition, dysbiosis of intestinal flora caused dysregulation of the linoleic and arachidonic acid metabolism, a decrease in prostaglandin derivatives, and an imbalance of anti-/pro-inflammatory fatty acids, all of which are potentially damaging factors to intestinal mucosal barrier function. Toxic metabolites, such as indoxyl-sulfate and trimethylamine N-oxide, entered and accumulated in the blood through the injured intestinal mucosa. More importantly, disruption of the intestinal tryptophan metabolic pathway decreased circulating 3-indolepropionic and indoleacrylic acids, and significantly increased kynurenine levels. 3-indolepropionic acid, indoleacrylic acid, and kynurenine synergistically managed the expression of Gd-IgA1, IL-22, and TNF-a.

## Discussion

Metabolites produced by gut microbes exert their role in the host as signaling molecules and substrates for metabolic reactions, which help maintain host-gut microbial system homeostasis. Previous studies have found that short-chain fatty acids, amino acid derivatives, and gut-derived metabolites, such as lipopolysaccharide and trimethylamine, were associated with inflammatory and immune diseases (26, 27). These metabolites stimulate intestinal mucosal immune cells to continuously create inflammatory factors that drive persistent systemic inflammatory responses in chronic kidney disease (27). The characteristics of lipid and amino acid metabolism in non-treated IgAN patients remain poorly understood.

Lipid metabolism is closely related to immunological and inflammatory responses *in vivo* (28). We first focused on the metabolic profile of intestinal lipids in IgAN patients. We revealed that IgAN patients had significantly reduced levels of intestinal unsaturated fatty acids and fatty acid derivatives. Unsaturated fatty acids have been shown to interfere with leukocyte migration, modulate macrophage phagocytosis and cellular immune factor production, and inhibit TLR-mediated pro-inflammatory signaling pathways activated by saturated fatty acids (29). Reduced levels of intestinal unsaturated fatty acids suggested a lower suppressive effect of intestinal immunity, which might cause immune hyperactivation in IgAN patients. Our results also revealed that abnormalities in the linoleic and arachidonic acid metabolic pathways were another essential feature of intestinal metabolism in IgAN, which led to a general decrease in levels of protective intestinal metabolites such as prostaglandin derivatives and epoxy fatty acids. Although the interrelationships between the reduction in prostaglandins and epoxy fatty acids and intestinal mucosal damage remained unclear, our findings suggested that the intestinal system of IgAN patients is experiencing a weakening of mucosal protective metabolites (30, 31). The dysregulation of anti-/pro-inflammatory metabolites in the gut deserves special attention. Increased levels of pro-inflammatory arachidonic acid metabolites, decreased levels of anti-inflammatory unsaturated fatty acids, and dysregulation of conjugated linoleic acid with immunomodulatory effects (e.g., alpha-stearic acid and punicic acid) may play a role in persistent intestinal inflammatory and immune activation in IgAN patients (32). In addition, short-chain fatty acids (e.g., acetic acid, propionic acid, and butyric acid), which exerted anti-oxidative and anti-inflammatory effects on the gut system, have been reported to be reduced in IgAN patients (33). However, we failed to identify these small-molecule compounds in our study, which could be due to the different conditions of the technique.

On the other hand, gut microbes can produce potentially hazardous amino acid derivatives (e.g., phenols, ammonia, and sulfur-containing compounds) by decomposing food-related proteins (34). We observed a trend toward an increase in the levels of free amino acids in the gut and blood circulation systems. Notably, De Angelis's study revealed that free amino acid levels increased with progressed disease stage of IgAN (35). In addition, we also found disturbances in metabolites from amino acid metabolism in IgAN patients, with the most remarkable disorders in aromatic tryptophan metabolism. Tryptophan has three metabolic pathways *in vivo* (36). We first observed changes in the intestinal tryptophan-indole metabolic pathway. Our results showed that most indole metabolites, such as indole-3-acetic acid and 3-indolepropionic acid were significantly down-regulated. These indole metabolites, the primary ligands of the AhR signaling, are essential components of the immunological barrier of the intestinal mucosa, effectively promoting the renewal of the intestinal mucosal epithelium and maintaining the integrity of the intestinal mucosa (37). Reduced intestinal 3-indolepropionic acid levels have been reported to weaken the intestinal barrier, increase gut permeability, and trigger inflammatory responses in the intestine (6, 38). More importantly, a decrease in intestinal 3-indolepropionic acid levels led to an increase in intestinal secretory IgA and IgG in Clostridium sporogenes-deficient mice (6), revealing an important role of 3-indolepropionic acid in immunoregulation. Notably, intestinal 3-indolepropionic acid deficiency directly led to a decrease in 3-indolepropionic acid in the blood. Decreased serum 3-indolepropionic acid concentrations were previously reported to be associated with an increase in circulating neutrophils and Ly6C+ monocytes, as well as CD4+ and CD8+ effector/memory T cells (6). Therefore, 3-indolepropionic acid may be an important mediator linking the immune communication between intestinal and blood circulation systems. We also revealed high levels of serum proinflammatory factors (TNF-a and IL-6) in IgAN patients, which have been demonstrated to be associated with renal impairments and the IgAN pathogenesis (39–41). Anti-inflammatory factors (IL-10 and IL-22) associated with mucosal immunity were also increased in IgAN patients, indicating a possible compensatory mechanism for the overactivation of the immuno-inflammatory response (42). Interestingly, 3-indolepropionic acid and kynurenine were strongly associated with the expression of Gd-IgA1, IL-22, and TNF-a in IgAN patients. We speculated that 3-indolepropionic acid synergistically managed the body's immunity with kynurenine because there is a strong correlation between these two metabolites (24, 43). Finally, we found that 3-indolepropionic acid also had high sensitivity and specificity in the diagnosis of IgAN. Sun's study has reported that gut-derived 3-indolepropionic acid might be a significant biomarker for CKD as well as a nephroprotective agent against CKD development (44). Animal studies and randomized clinical trials that confirm the clinical utility of 3-indolepropionic acid in IgAN remain for future research.

## Conclusion

Our results showed that IgAN patients have significant disturbances of fatty acid and aromatic amino acid metabolism, particularly linoleic acid, arachidonic acid, and tryptophan metabolism. Changes in these metabolites revealed disorders of the anti-/pro-inflammatory microenvironment and attenuated intestinal mucosa immune in the gut. Decreased levels of circulating 3-indolepropionic acid caused by disruption of the intestinal tryptophan metabolic pathway can promote the expression of Gd-IgA1, IL-22, and TNF-a.

## Data Availability Statement

The datasets presented in this study can be found in online repositories. The names of the repository/repositories and accession number(s) can be found below: NCBI; PRJNA785415. Other raw data supporting the conclusions of this article will be made available by the authors.

## Ethics Statement

The present study was conducted following the principles of the Declaration of Helsinki and was authorized by the Ethics Board of Shenzhen People's Hospital (LL-KY-2019514). The patients/participants provided their written informed consent to participate in this study.

## Author Contributions

YD, LY, and FL designed the experiments. HW, MY, and DT performed the experiments and wrote the manuscript. HW, HL, and SH performed the computational analysis. CY and XZ collected and assembled data. BH provided writing and language assistance. All authors critically revised the manuscript.

## Funding

This work was funded by Guangdong Engineering Technology Research Center (Grant Number: 507204531040); Guangzhou Development Zone entrepreneurship leading talent project (No: 2017-L153); Guangdong province union training postgraduate demonstration base (No: 20190630); Guangzhou entrepreneurship leading team (No: 202009030005); Shenzhen Fund for Guangdong Provincial High-level Clinical Key Specialties (No: SZGSP001).

## Conflict of Interest

The authors declare that the research was conducted in the absence of any commercial or financial relationships that could be construed as a potential conflict of interest.

## Publisher's Note

All claims expressed in this article are solely those of the authors and do not necessarily represent those of their affiliated organizations, or those of the publisher, the editors and the reviewers. Any product that may be evaluated in this article, or claim that may be made by its manufacturer, is not guaranteed or endorsed by the publisher.
