# Supplementary material for: Metabolic Dysfunctions of Intestinal Fatty Acids and Tryptophan Reveal Immuno-Inflammatory Response Activation in IgA Nephropathy
Source: Front Med (Lausanne). 2022 Feb 3;9:811526. doi: 10.3389/fmed.2022.811526 (PMC8850467; doi:10.3389/fmed.2022.811526)
Supplement: Supplementary file 11 [file Data_Sheet_1.DOCX]

**
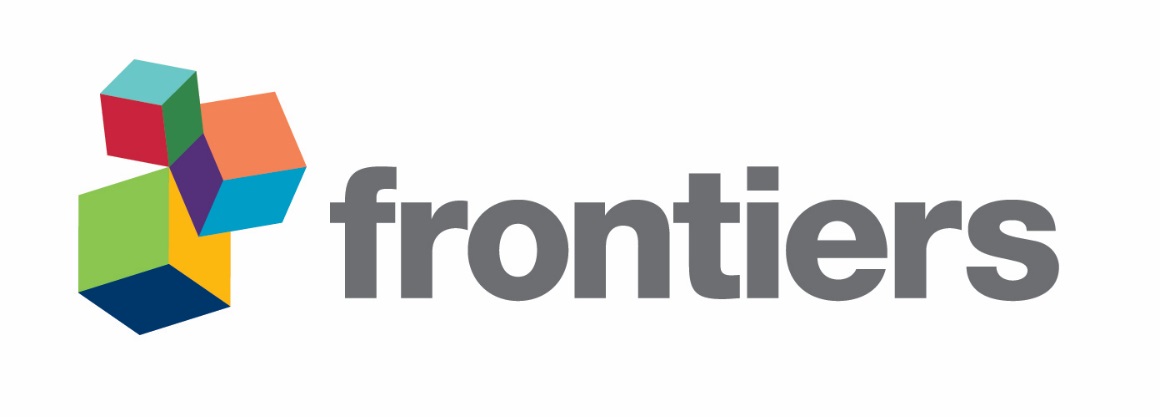
**

**Supplementary Materials**

Supplementary Figure 1: Overall changes in fecal metabolites in IgAN.

Supplementary Figure 2: Comparison of the peak intensity of the intestinal lipid metabolites in the IgAN and HC groups.

Supplementary Figure 3: Metabolic characteristics of the blood circulatory system in IgAN patients.

Supplementary Figure 4: Alterations in circulating tryptophan metabolic pathways in IgAN.

Supplementary Figure 5. Network diagram depicting Spearman’s correlations between between derived secondary metabolites and immune-inflammatory factors.

Supplementary Table 1: Characteristics of the included subjects.

Supplementary Table 2: Quantitative and calibration of tryptophan metabolites.

Supplementary Table 3: Classification of identified metabolites based on ChEBI Ontology.

Supplementary Table 4: Significantly changed metabolites in fecal samples of IgAN patients.

Supplementary Table 5: Enrichment analysis of altered metabolite class by Fisher's exact test.

Supplementary Table 6: Spearman's analysis of significantly altered lipids in fecal samples.

Supplementary Table 7: Significantly changed metabolites of serum samples in IgAN patients.

Supplementary Table 8: Changes in serum tryptophan metabolites in IgAN patients.

Supplementary Table 9: Spearman's analysis of metabolites in the tryptophan pathway.

Supplementary Table 10: Correlation network for the intestinal flora, serum metabolites, and fecal metabolites in IgAN patients.


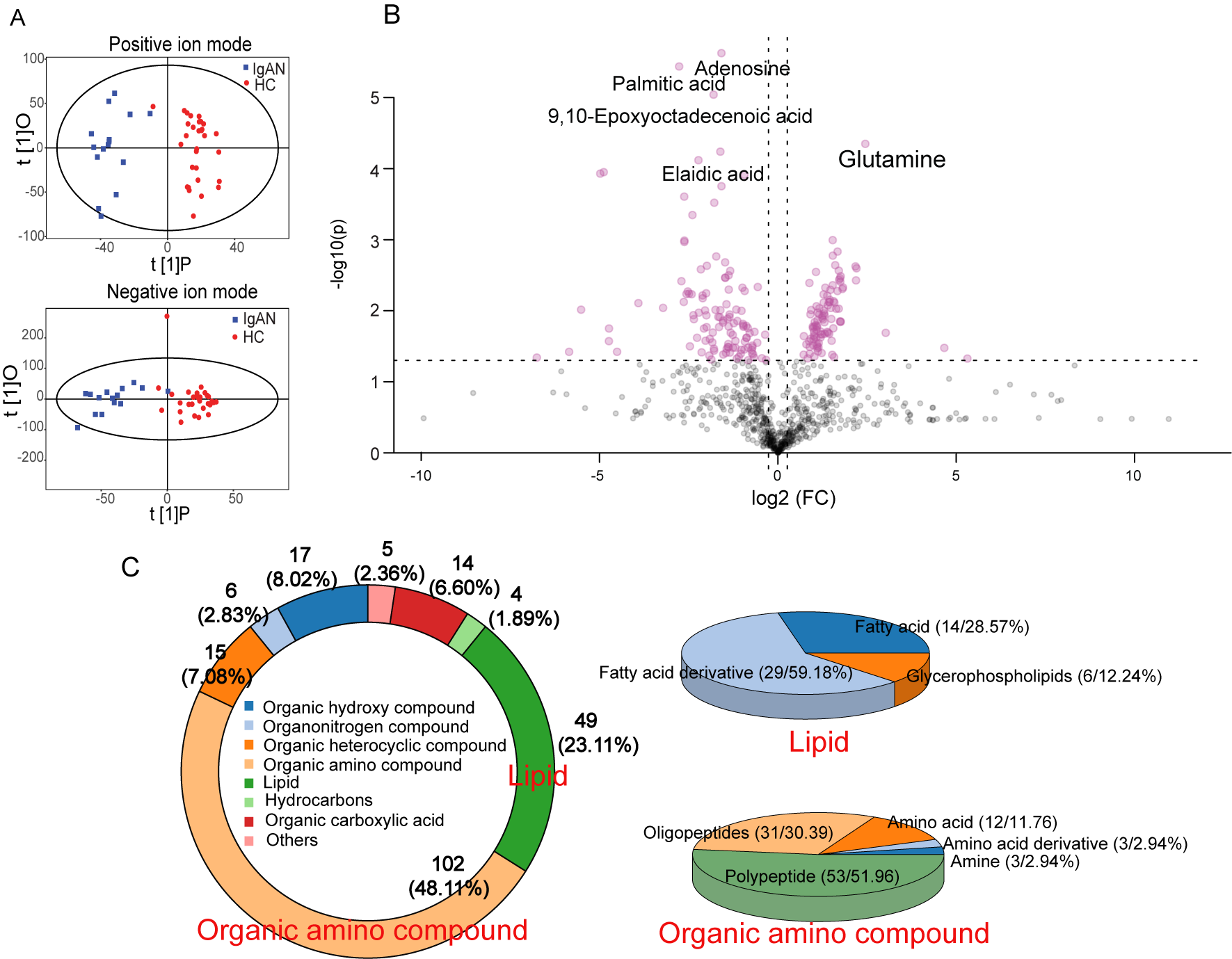


Supplementary Figure 1. Overall changes in fecal metabolites in IgAN. (A) Construction of the OPLS-DA model of fecal metabolites. Scatter plot of OPLS-DA model scores in the IgAN and HC groups. The horizontal coordinate t[1]P indicates the predicted principal component score of the first principal component, while the vertical coordinate t[1]O represents the orthogonal principal component score. (B) Volcano plot showing the significantly changed metabolites in feces samples of IgAN patients. (C) Classification features and proportion of the secondary metabolites in feces sapmes based on the ChEBI Ontology metabolite database.


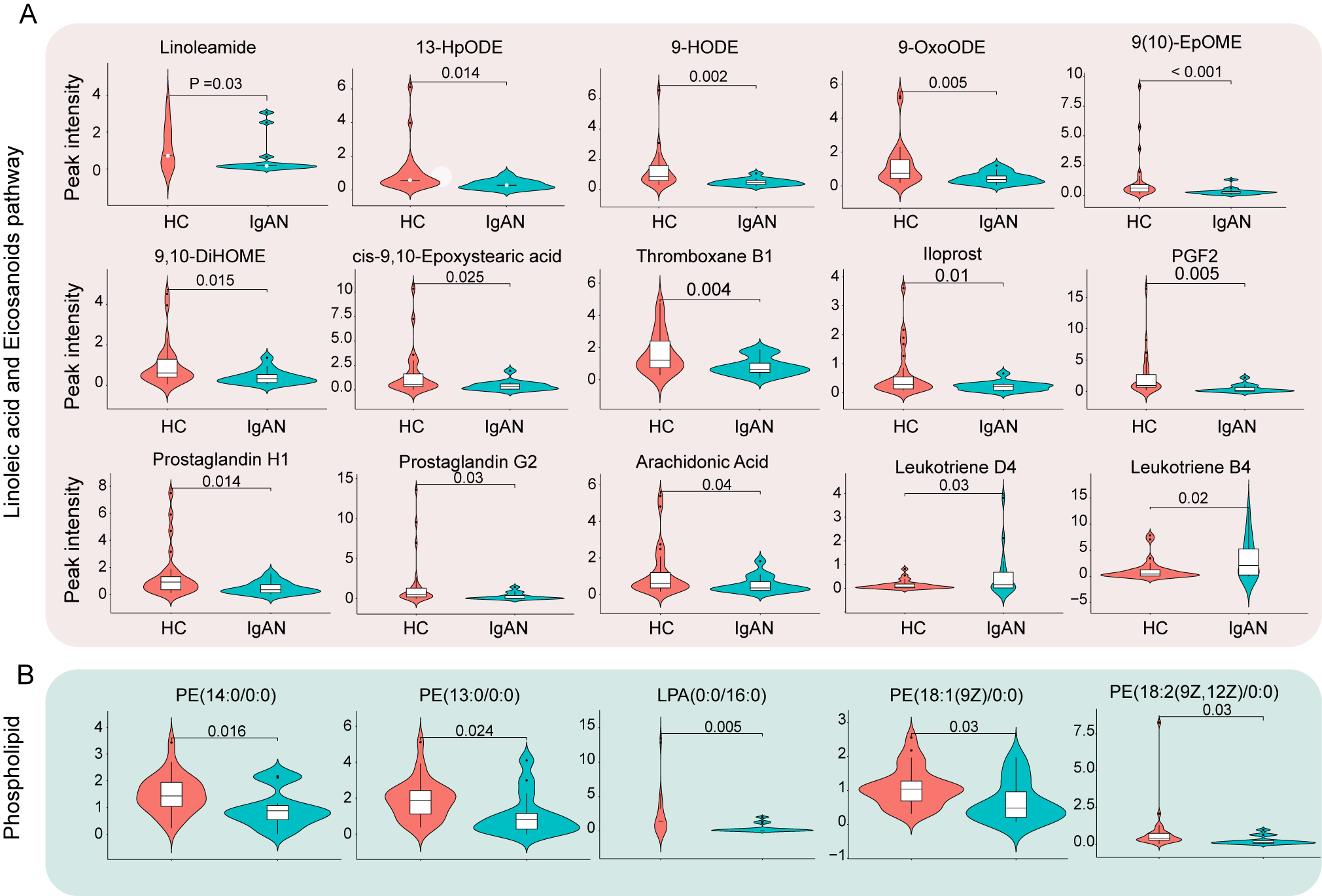


Supplementary figure 2. Comparison of the peak intensity of the intestinal lipid metabolites in the IgAN and HC groups. (A) The peak intensity of the metabolites of the linoleic acid and eicosanoids metabolism in the feces samples. (B) The peak intensity of different types of phospholipids in the feces samples.


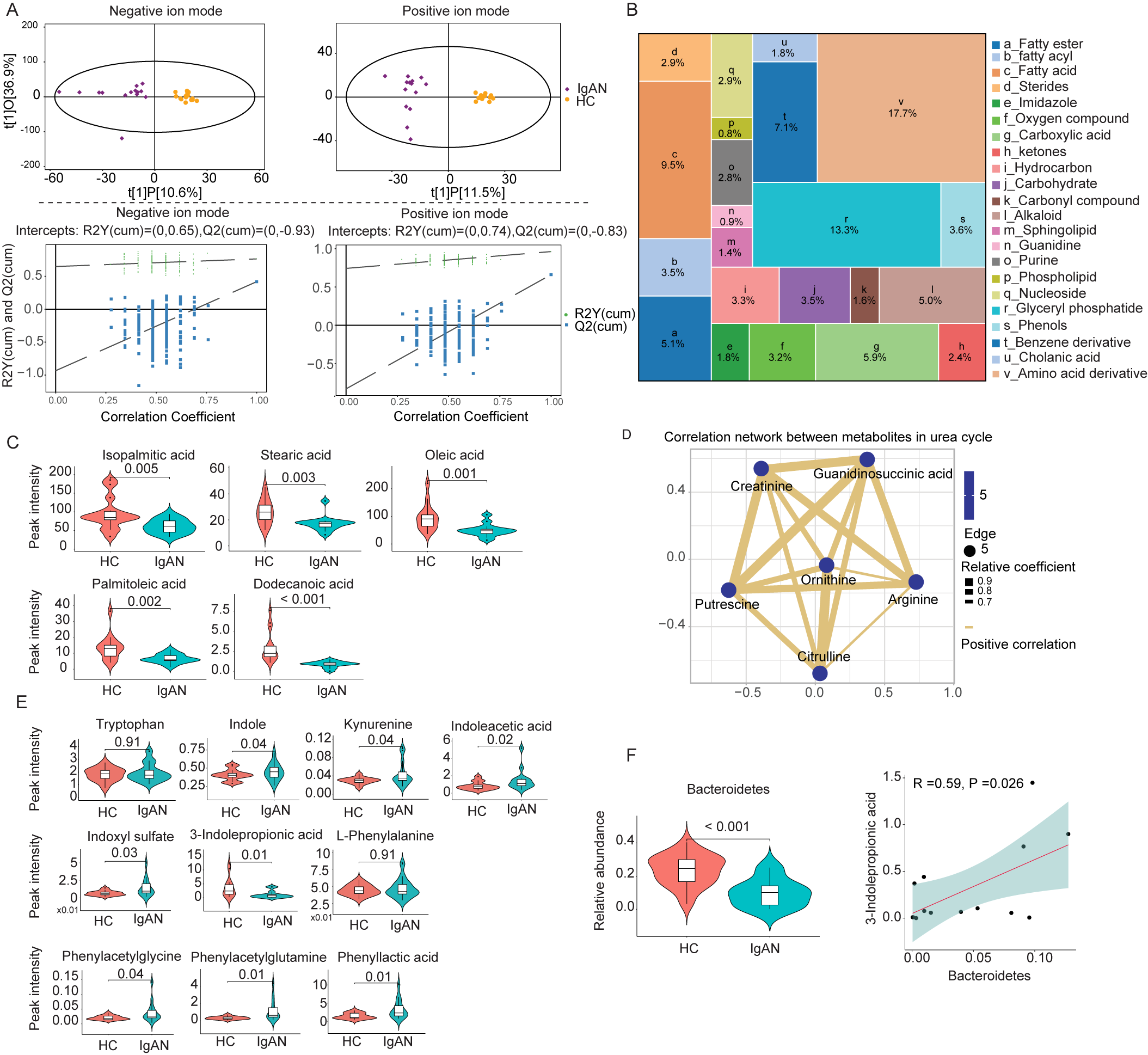
Supplementary Figure 3. Metabolic characteristics of the blood circulatory system in IgAN patients. (A) Construction of the OPLS-DA model of serum metabolites. Up: scatter plot of OPLS-DA model scores in the IgAN and HC groups. Down: results of the permutation test of the OPLS-DA model. R2 indicates the degree to which the variation in each variable is explained. The Q2 intercept value represents the robustness of the model, and risk of overfitting and the reliability of the model. (B) Classification features of the serum secondary metabolites based on the ChEBI Ontology metabolite database. Different metabolite classes are represented by distinct colors and letters. The percentage indicates the number of metabolites in that classification as a proportion of the total number of metabolites. (C) The peak intensity of serum anti-inflammatory lipids in the IgAN and HC groups. (D) Correlation network between metabolites in the urea cycle. (E) The peak intensity of enteric-derived metabolites generated through tryptophan and phenylalanine metabolism in serum samples. (F) The relationship between 3-indolepropionic acid and intestinal Bacteroidetes.


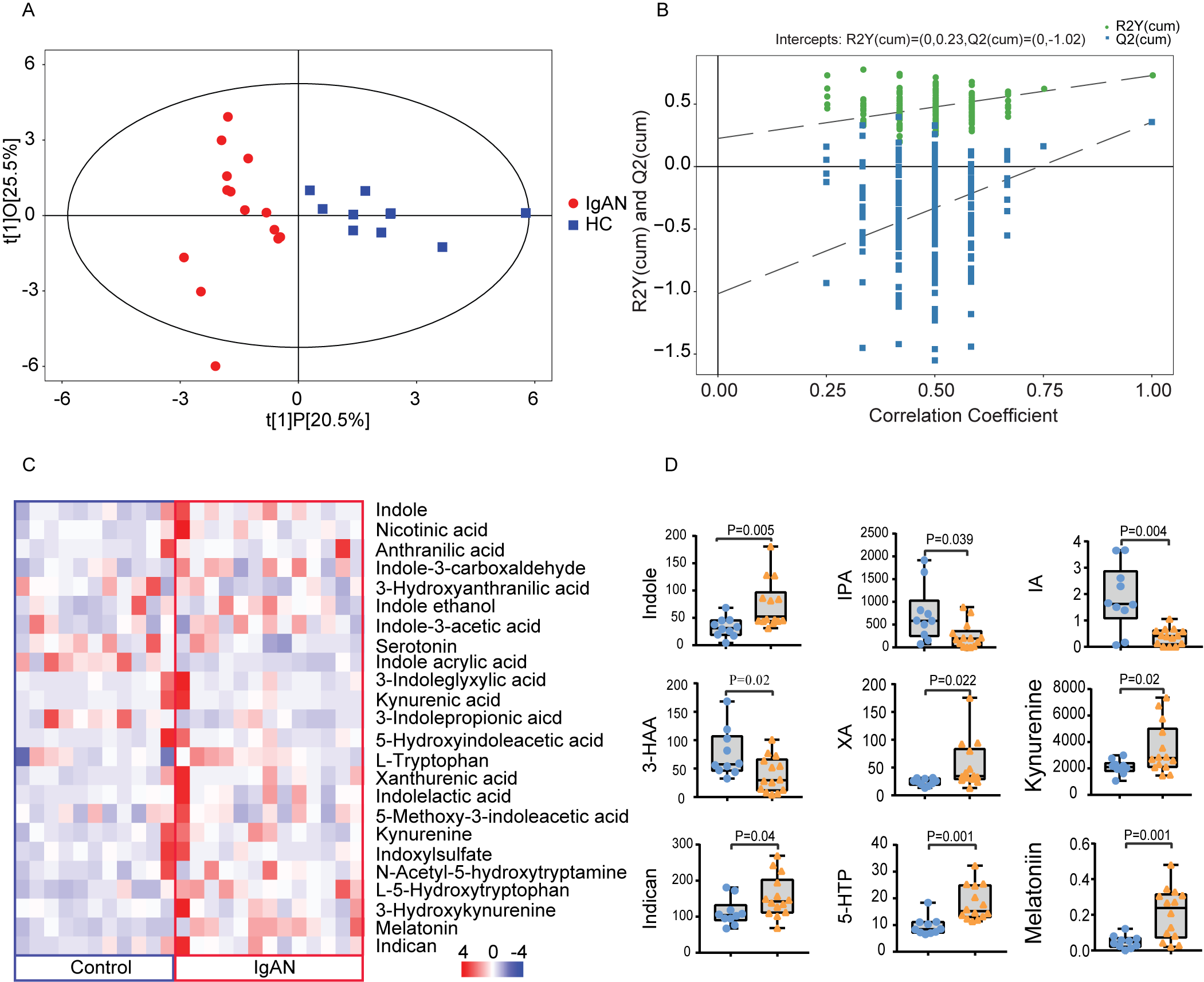


Supplementary Figure 4. Alterations in circulating tryptophan metabolic pathways in IgAN. (A) Construction of the OPLS-DA model of serum metabolites of tryptophan metabolism. (B) Results of the permutation test of the OPLS-DA model. (C) Heatmap showing the [absolute](D:/software/Dict/8.9.8.0/resultui/html/index.html#/javascript:;) [abundance](D:/software/Dict/8.9.8.0/resultui/html/index.html#/javascript:;) of the 24 tryptophan metabolites in the IgAN and HC groups. (D) Box-plots depicting significantly altered tryptophan metabolites in IgAN patients.
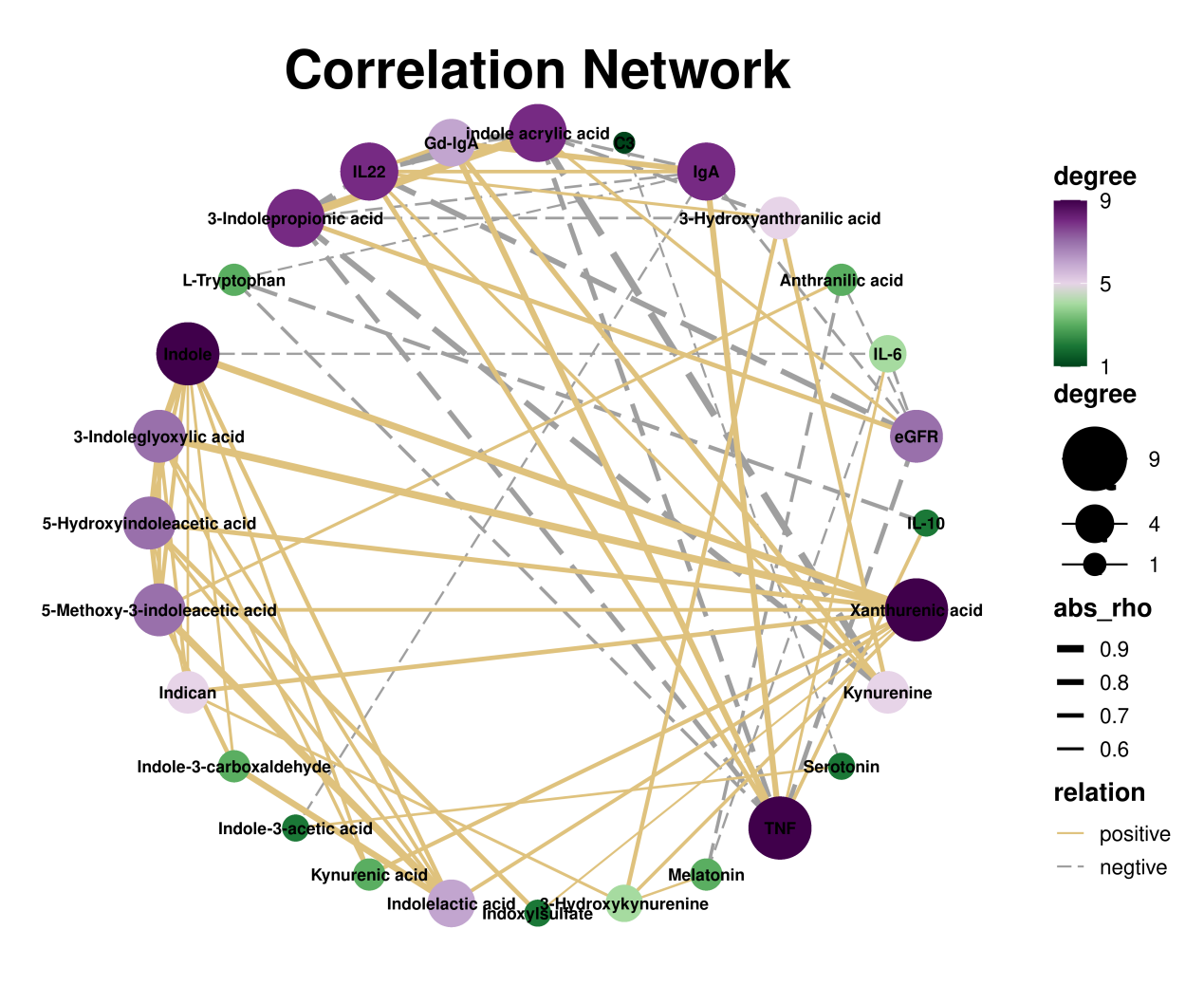


Supplementary Figure 5. Network diagram depicting Spearman’s correlations between between derived secondary metabolites and immune-inflammatory factors. The solid yellow line indicates positive correlations, whereas the dashed blue line represents negative correlations. Thicker lines refer to stronger relationships. Pairs with pearman’s coefficient > 0.6 and P-value < 0.05 are shown in the network.
